# Audit on structure of assessment for remote consultation during COVID-19 pandemic

**DOI:** 10.1192/bjo.2021.234

**Published:** 2021-06-18

**Authors:** Astha Das, Margaret Kuklewicz

**Affiliations:** Nottinghamshire Healthcare NHS Trust

## Abstract

**Aims:**

According to the Royal College of Psychiatry, GMC guidelines and NHS England, it is necessary to consider remote consultation to enable service delivery to those requiring shielding or facing additional health risk, and to avoid transition of infection.

To audit whether the standards of Mobile and Remote access work are met.

To audit whether the standards of Consent to Examination and Treatment are met.

To also evaluate whether the remote consultation due to the COVID-19 pandemic is being explicitly documented or not.

To suggest to the policy makers the need to establish some standards of practice concerning remote consultation and consent in the COVID-19 pandemic

**Method:**

Inclusion criteria – sample of service users who had remote consultation in April, May, and mid-June 2020 by doctors of MHSOP community mental health team at Bassetlaw Hospital.

Data collection: Retrospective.

Data source(s) used: Patient/Client medical/care records

Anticipated benefits of this audit: Due to the nature of current COVID-19 pandemic situation, it is essential to minimise contacts with vulnerable groups to prevent transmission of infection. It is anticipated that the number of remote consultations will grow in the forthcoming months.

This audit creates an opportunity to develop a new policy and improve the quality of remote consultations documentation.

**Result:**

Documentation for remote consultation was done in 81% of case notes whereas documentation of consent obtained was present in 57% of patients’ electronic notes.

90% of entries had documentation of ‘addressed concerns’. Around 50-70% of patients’ documents showed good record keeping on domains of ‘ability to maintain effective communication’, ‘mental state examination’, ‘risk assessment’ and ‘ability to understand medication plus side effects’.

About 40% of documentation met standards for good record keeping on ‘management plan’, ‘concerns raised’, ‘chance given to ask about management plan’.

**Conclusion:**

Most of the standards of good consultations are being met despite the change in the type of Consultation due to COVID-19. However, there are identified areas for improvement which could be focused on. For example, documentation can be clearer when consent is gained for remote consultation. It should not be presumed that, as patients are booked in a certain type of clinic, they have been properly consented beforehand.

Key Success: Almost in all domains 40% have met the standards

Key Concerns: There are areas where a lot of evidence is partially documented.

The above results can be explained as a consequence of a sudden change in the normal working pattern in a community-based setting, having minimal protocols and procedures on standards of working in the situation of COVID19 remote consultation.

Following this audit, we aim to increase the amount of information recorded during remote consultation.

The plan is to develop a template that would cover the requirements for a remote consultation recommended by national guidelines

The proposal of a letter template following a remote consultation will be disseminated to the MHSOP CMHT teams for any suggestions/approval.

